# Microplastics in snow from protected areas in Hokkaido, the northern island of Japan

**DOI:** 10.1038/s41598-023-37049-5

**Published:** 2023-06-19

**Authors:** Hiroshi Ohno, Yoshinori Iizuka

**Affiliations:** 1 Kitami Institute of Technology, Kitami, Hokkaido Japan; 2 Institute of Low Temperature Science, Hokkaido University, Sapporo, Hokkaido Japan

**Keywords:** Environmental sciences, Cryospheric science

## Abstract

Snowfall is regarded as a carrier of airborne microplastics (MPs). Deposited snow can function as a temporary reservoir for atmospheric MPs. Nevertheless, knowledge and understanding of MPs in snow remain sparse. This study investigates the abundance, composition, size (> 30 µm), and shape of MPs in snow specimens from various nature preservation areas and also from urban sites in Hokkaido. Various polymeric-type MPs with mostly fragmentary shapes were detected among the specimens. More than half of MPs were in the smallest size class (30–60 µm), implying the presence of more MPs below the limit (< 30 µm). Concentrations of MPs ranged from 1.5 × 10^2^ to 4.2 × 10^3^ particles/L. The results demonstrated that microplastic abundance generally decreases concomitantly with increasing remoteness of sampling sites. Observed features of MPs at different locations and their relation to geographical settings have indicated that the ubiquitously observed fine particles (mainly alkyd, ethylene–vinyl acetate, and polyethylene) are attributable to long-distance atmospheric transportation, whereas the rubber and larger particles especially found near highways and cities are from local sources of plastic. Taken together, these findings suggest important implications for elucidating the nature and distribution of atmospheric MPs.

## Introduction

Since considerable microplastic pollution in marine sediment and plankton samples was reported in 2004 by Thompson et al*.*^1^, increasing evidence has accumulated to suggest that MPs are ubiquitous pollutants on a global scale, from the deep sea floor^2^ to the top of the world (Mt. Everest)^3^. Uptake and accumulation of MPs and their related chemicals in organisms are regarded as hazardous for ecological systems^4,5^ and for human health^6^. Moreover, recent studies have emphasized the possible impact of microplastics on global carbon and nitrogen cycles^7,8^.

Microplastics in marine and freshwater environments have been studied extensively in terms of their distribution, sources, and transportation^9–11^. By contrast, airborne microplastics have been investigated less intensively, although the importance of atmospheric transportation of MPs to remote areas has been underlined^12–17^. Microplastics can be captured in clouds during their formation processes^18^ and can be scavenged by subsequent precipitation^13^. Snowfall is therefore regarded as a factor controlling the spatial distribution of MPs. Importantly, snow deposited on the Earth surface is regarded as a temporary sink for microplastics of both dry and wet depositions, providing opportunities to investigate atmospheric MPs^19^.

Pioneering research conducted by Bergman and others^14^ brought to light the presence of great numbers of fine microplastics in snow from Europe and the Arctic, and highlighted atmospheric transport and deposition as notable pathways for MPs meriting more research. Subsequently, several studies have confirmed that microplastic pollution in snow spreads worldwide^3,17,20–28^. Nevertheless, because of limited data, little is known about the features and pathways of microplastics in snow. To elucidate the nature and origin of microplastics in snow, we investigated the abundance, polymer type, size, and shape of MPs in snow from protected areas with different geographical settings and also from urban sites in Hokkaido, the northern island of Japan, using techniques based on microscopy and micro-Fourier-Transform Infrared Spectroscopy (micro-FTIR).

## Methods

### Study sites

**Figure 1:**
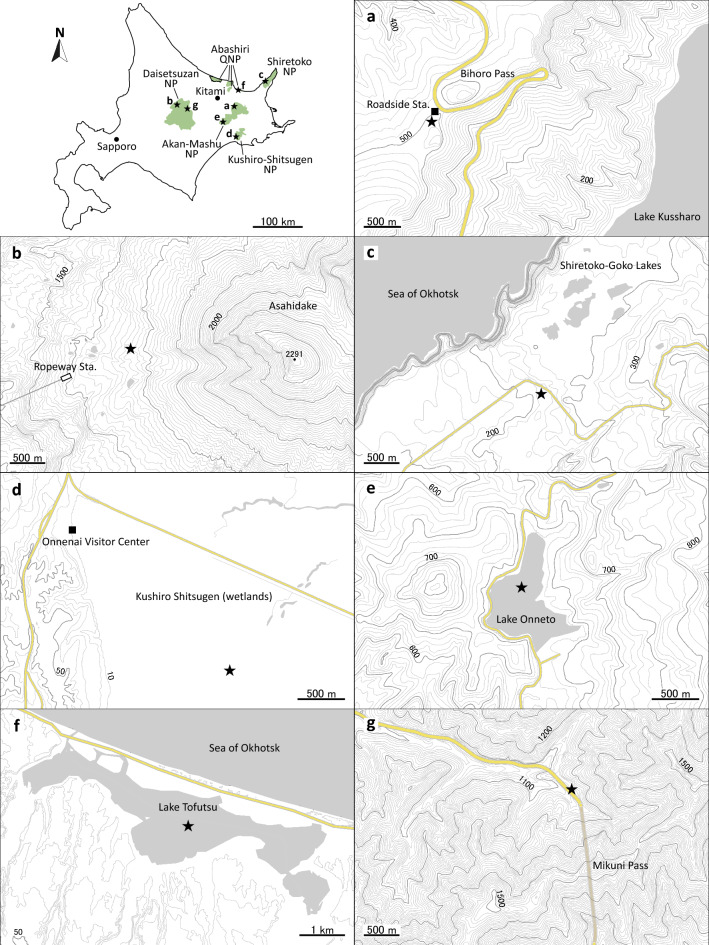
Locations of snow-sampling sites (solid stars) in Hokkaido, the northern island of Japan. Maps were created using ESRI ArcGIS Pro (ver. 3.0.3, https://www.esrij.com/products/arcgis-pro/). Topographic data were from Fundermental Geographic Data published by Geospatial Information Authority of Japan.

The Bihoro Pass in the Akan-Mashu National Park (NP) is located on the western rim of the Kussharo Caldera (Fig. 1a). Near the sampling point, a roadside station that is open year-round is accessible by automobile. Another site in the Akan-Mashu NP is Lake Onneto (Fig. 1e), which has a reputation for its amazing colored waters and majestic mountain landscapes. A roadway leading to the lake is closed in winter, but the lake and its surroundings are popular spots for snowshoe hikers. Asahidake, the highest peak in Hokkaido, is a part of the Daisetsuzan NP (Fig. 1b). A ropeway station halfway up the mountain is crowded with skiers and snowboarders on weekends. However, almost nobody accesses the area around the sampling point, approximately 1000 m distant from the station. Another site in the Daisetsuzan NP is Mikuni Pass (Fig. 1g): the highest section among all national routes in Hokkaido. The sampling point is the top of a berm beside the national route (open year-round). Shiretoko-Goko Lakes in the Shiretoko NP are small five lakes surrounded by native forest (Fig. 1c). Shiretoko is designated as a UNESCO World Natural Heritage Site because of its interactive marine and terrestrial ecosystems. During winter, access to the lakes is limited by road closure. The lakes are open only to small groups of tourists. The Kushiro-Shitsugen NP (Fig. 1d), which has the largest wetland area in Japan, is protected under the Ramsar Convention. Lake Tofutsu (Fig. 1f), a part of the Abashiri Quasi-National Park (QNP), is a brackish lake known as a Ramsar sanctuary for wild birds. Visitors are rare at these Ramsar sites, particularly in winter. For comparison, two urban sites were investigated in Kitami, which is the largest city in Okhotsk sub-prefecture, with population of approximately 110,000. One site is a tennis court on the campus of Kitami Institute of Technology (Kitami_KIT). The other is a football field on a riverbed of the Tokoro River (Kitami_RB). Both urban sites are closed during the winter.

### Snow sampling

Snow deposited on the ground (or on a frozen lake) was collected during sampling. All samples were pristine-white snow: no dirt was visible to the naked eye. The surface snow, approximately the upper 5 cm, was transferred with a stainless-steel spoon from snow deposited into a glass bottle. Then a metal lid was screwed on the bottle. Sampling was conducted always downwind using bare hands.

### Sample preparation

Snow samples were thawed in closed glass bottles at room temperature. Approximately 200 mL of each thawed sample (all water in the bottle) was filtered onto an aluminum oxide filter (25 mm diameter, 0.2 µm pore size, Anodisc; Whatman)^29^ using a vacuum filtration apparatus made of glass. With the filter still attached to the apparatus, approximately 30 mL of 30% H_2_O_2_ was poured into a glass funnel of the apparatus. The opening of the funnel was covered with aluminum foil. The solution was then left for 1 day at room temperature to remove natural organic matter adhered to MPs^30^. Then the solution was filtered with the apparatus. After finishing the filtration of approximately 50 mL of ultra-pure water (18 MΩ cm), the filter was removed from the apparatus and was dried in a glass petri dish. All processes described above were performed in a clean booth with a HEPA filtration unit.

As replicates, spare samples collected from the same snow deposits were investigated for Asahidake, Mikuni Pass, and Kitami_RB, whereas one sample per location was measured for other study sites.

### Microscopic observation

**Figure 2:**
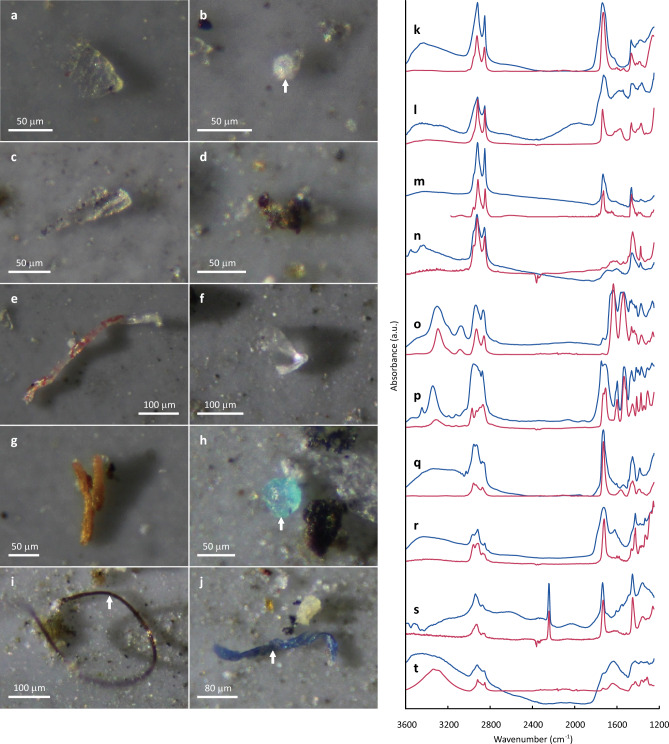
Micrographs and FTIR spectra of particles from snow samples identified as (**a**,**k**) alkyd, (**b**,**l**) ethylene–vinyl acetate, (**c**,**m**) polyethylene, (**d**,**n**) rubber, (**e**,**o**) polyamide, (**f**,**p**) polyurethane, (**g**,**q**) polymethyl methacrylate, (**h**,**r**) polyvinyl chloride, (**i**,**s**) polyacrylonitrile, and (**j**,**t**) rayon. Spectra from the particles are shown with blue lines, whereas those for references^31–34^ are presented as red lines.

After a prepared filter was placed in an optical petri dish made of quartz, it was inspected through a quartz optical window using an optical microscope (BHT; Olympus Corp.). To enhance the visibility of microparticles on a filter, the filter surface was illuminated laterally by white LED light from one side. For this study, only particles larger than 30 µm were examined. We randomly selected 100 fields of view with dimensions of 0.84 × 1.27 mm^2^, and counted particles in each field of view. From the average number of particles in the field of view and the filtration area (224.5 mm^2^), the numbers of particles on a filter were estimated (Table 1). Additionally, after randomly selecting approximately 100 particles for each filter as targets of micro-FTIR analysis, we recorded their coordinate positions on filters. The particles were classified by shape, as having fragments or fibers. Targeted particles were photographed using a digital camera (EOS Kiss X3; Canon Inc.) attached to the microscope. Then their maximum diameters or lengths were measured by analyzing micrographs using image-processing software (ImageJ; NIH). Micrographs of typical particles are presented in Fig. 2a–j.

**Table 1:** Altitudes of study sites, collection dates of snow samples, filtered volumes of melted snow samples, the numbers of particles (> 30 µm) on filters, and abundances of MPs (> 30 µm).

Sample code	Altitude (m a.s.l.)	Collection date	Filtered vol (mL)	Number of particles (particles/filter)	Microplastic abundance (particles/L)
Bihoro Pass	505	20 Mar. 2021	139.4	1.6 × 10^3^	2.0 × 10^3^
Asahidake [1]	1674	23 Mar. 2022	147.9	3.8 × 10^2^	1.7 × 10^2^
Asahidake [2]			171.5	4.3 × 10^2^	1.5 × 10^2^
Shiretoko-Goko Lakes	216	13 Jan. 2023	200.3	4.4 × 10^2^	1.9 × 10^2^
Kushiro Shitsugen	5	17 Jan. 2023	173.4	7.2 × 10^2^	5.0 × 10^2^
Lake Onneto	640	22 Jan. 2023	208.6	1.2 × 10^3^	7.4 × 10^2^
Lake Tofutsu	1	04 Feb. 2023	177.8	6.4 × 10^2^	3.1 × 10^2^
Mikuni Pass [1]	1143	05 Feb. 2023	159.8	8.3 × 10^2^	9.6 × 10^2^
Mikuni Pass [2]			153.6	8.0 × 10^2^	8.2 × 10^2^
Kitami_KIT	93	09 Jan. 2021	182.0	1.3 × 10^3^	1.4 × 10^3^
Kitami_RB [1]	57	19 Jan. 2023	233.1	4.3 × 10^3^	4.0 × 10^3^
Kitami_RB [2]			218.4	4.5 × 10^3^	4.2 × 10^3^

Numbers in square brackets signify replicates.

### Micro-FTIR analysis

Chemical identifications of microparticles were done using micro-FTIR. All targeted particles on filters were analyzed using an FTIR microscope (Nicolet iN10; Thermo Fisher Scientific) in transmission mode with the following parameters: 30 μm × 30 μm square field aperture, 8 cm^−1^ spectral resolution, 64 scans, and 1250–3600 cm^−1^ spectral range. The obtained FTIR spectra were then compared with the commercial spectral databases of standard polymers (Hummel Polymer sample Library and HR Polymer and additives) and also with open-access libraries designed for microplastic research, which includes spectra of aged plastics^31–34^, using spectroscopy software (OMNIC Picta; Thermo Fisher Scientific). Spectra with a match of < 60% were rejected. When returning a spectral match of > 60%, an additional visual examination of spectra was performed manually, leading to final acceptance or rejection^35–37^. FTIR spectra of typical particles are presented in Fig. 2k–t. Although some anthropogenic cellulosic fibers such as rayon were detected (e.g. Fig. 2j,t), these are not classified as plastics but as celluloses for these analyses.

### Blank test

To examine the validity of methods, blank tests were conducted. During snow sampling, an uncovered empty glass bottle was left on the surface of the snow deposit for several minutes; then it was closed with a lid. After they were brought back to the laboratory, the blank bottle was filled with ultra-pure water. It was processed using the same procedures as those used for the snow samples. In all, three blanks were tested. Observations of blank-filter surfaces with the microscope revealed several particles on all blank samples. Subsequent micro-FTIR analyses of these particles detected three epoxy (or phenoxy) resins from the first blank, one cellulose and three epoxy (or phenoxy) resins from the second one, and one epoxy (or phenoxy) resin and one polystyrene from the last one. Inspection of “brand-new” filters revealed that a few epoxy (or phenoxy) resin particles were located on the filter surface, irrespective of the production lot. Therefore, two resin particles with features similar to those of the inherent particles found during particle analyses of the snow samples (*n* = 1276) were not counted as MPs from the snow.

## Results

### Microplastic abundances at different locations

In fact, microplastics were detected at all locations (Supplementary Information). Abundances of microplastics in snow samples were estimated from the percentages of identified MPs among the total particles analyzed (Fig. S1), the numbers of particles on filters, and the volumes of water samples (Table 1). The microplastic concentrations were approximately 2.0 × 10^3^ particles/L at Bihoro Pass, 1.7 × 10^2^ particles/L at Asahidake [1], 1.5 × 10^2^ particles/L at Asahidake [2], 1.9 × 10^2^ particles/L at Shiretoko-Goko Lakes, 5.0 × 10^2^ particles/L at Kushiro Shitsugen, 7.4 × 10^2^ particles/L at Onneto Lake, 3.1 × 10^2^ particles/L at Tofutsu Lake, 9.6 × 10^2^ particles/L at Mikuni Pass [1], 8.2 × 10^2^ particles/L at Mikuni Pass [2], 1.4 × 10^3^ particles/L at Kitami_KIT, 4.0 × 10^3^ particles/L at Kitami_RB [1], and 4.2 × 10^3^ particles/L at Kitami_RB [2] (Table 1).

### Microplastic compositions and sizes at different locations

**Figure 3:**
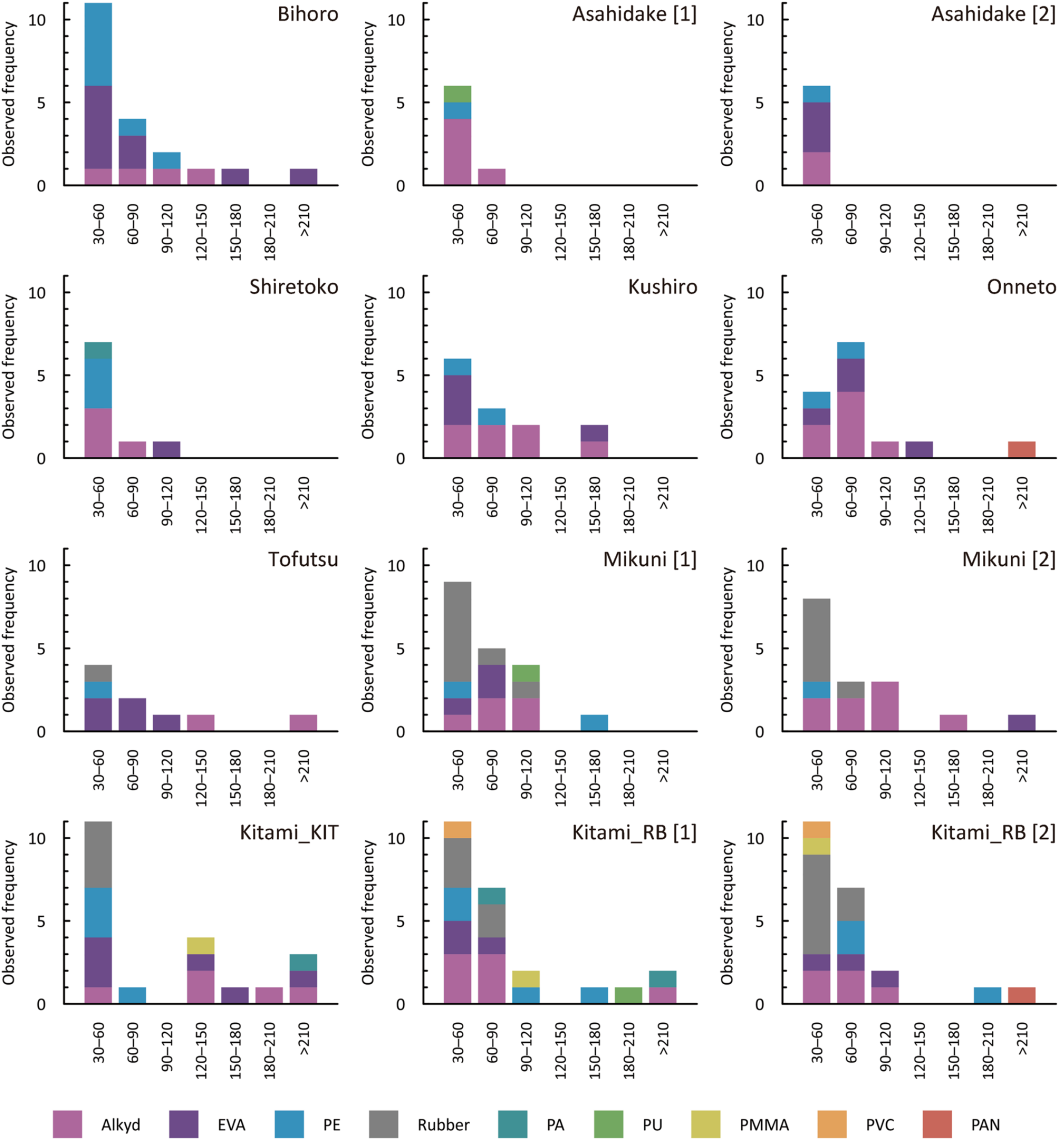
Observed frequencies of microplastics of various types from snow samples in different size classes (µm) at different locations.

**Figure 4:**
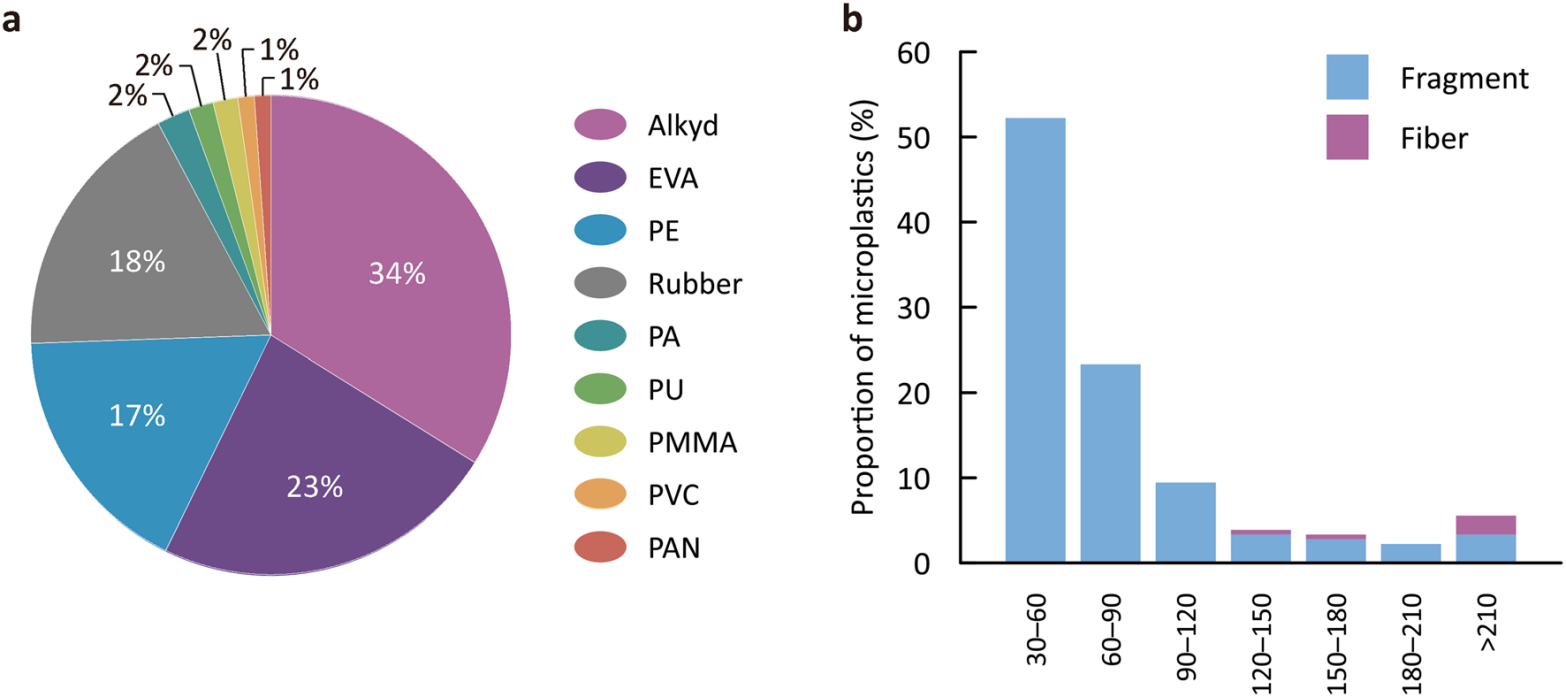
Data of microplastics from all snow samples (*n* = 180). (**a**) Polymer compositions of MPs. (**b**) Size distributions of fragment and fiber MPs (µm).

Observed frequencies of microplastics of various types from snow samples in different size classes are portrayed in Fig. 3. Nine types of plastic polymers were detected from microparticles: alkyd, ethylene–vinyl acetate (EVA), polyethylene (PE), rubber, polyamide (PA), polyurethane (PU), polymethyl methacrylate (PMMA), polyvinyl chloride (PVC), and polyacrylonitrile (PAN). Polymer compositions of MPs are summarized in Fig. 4a. Alkyd, EVA, PE, and rubber were the dominant compositions of MPs in the snow samples, accounting for 92%. Alkyd, EVA, and PE particles were observable almost anywhere at the study sites, whereas rubber particles were detected mostly at Mikuni Pass, Kitami_KIT, and Kitami_RB (Fig. 3).

At all locations, MPs were mainly in the 30–120 µm size range in maximum diameter or length (Fig. 3). As a general trend, most PE and rubber particles were of the size class below 90–120 µm, whereas alkyd and EVA particles were distributed throughout wide size ranges. Size distributions of fragment and fiber microplastics are presented in Fig. 4b. The shapes of MPs from snow samples were mostly fragments, accounting for 97%. More than 80% of fragment MPs were smaller than 120 µm in maximum diameter, whereas all fiber MPs were longer than 120 µm long. Fragment MPs consisted of all types of plastic polymers except for PAN, whereas fiber MPs comprised of alkyd, PA, and PAN.

## Discussion

Overall, the observed differences in microplastic abundance between sampling sites can be attributed to the degree of remoteness. Except for Bihoro Pass, the microplastic concentrations of snow samples from protected areas were an order of magnitude lower than those of urban samples (Table 1). High microplastic abundance at Bihoro Pass possibly occurs because of nearby human activities: the Bihoro Pass sampling site is located just beside a footpath that extends from the roadside station to a lookout. Asahidake and Shiretoko-Goko Lakes are regarded as the most remote places among our study sites. The lowest concentrations of MPs were detected at these locations, as expected.

Automobile traffic can be a main cause of localized observations of rubber particles at Mikuni Pass, Kitami_KIT, and Kitami_RB (Fig. 3). Results of earlier studies have indicated that, in traffic-related abrasion particles, tire wear and tear contributes significantly to the flux of microplastics into the environment^38,39^. Reportedly, tire and bitumen wear particles from urban streets are major sources of microplastics^40^. Kitami_KIT and Kitami_RB are urban sites. Although Mikuni Pass is located far from any town, snow was sampled near the national route (Fig. 1g). Most rubber particles found from snow samples were blackish (e.g. Fig. 2d), suggesting a black-tire-rubber origin.

No major source of plastic exists near the Asahidake site. Moreover, this remote site is located at a higher altitude than the atmospheric boundary layer (Table 1). The observed MPs were mostly in the smallest size class of 30–60 µm (Fig. 3). Considering these findings, these MPs are regarded as deriving from long-distance atmospheric transportation^12–17^. It is noteworthy that microplastic particles similar in size and also in composition (mainly alkyd, EVA, and PE) were observable at all locations (Fig. 3), implying that some of these fine MPs were from remote areas. A regional atmospheric transport model for microplastics (> 10 μm) over Asia and adjacent oceans recently developed by Long et al*.*^41^ has suggested that microplastic lines and fragments are efficiently transported > 1000 km because of their larger surface-area-to-volume ratios and lower densities than those of mineral dust particles. However, large MPs (> 120 µm) particularly found at the urban sites (Fig. 3) were probably from local sources of plastic^3,19,42^.

The features of microplastics in snow reported to date differ considerably among the reported descriptions^3,17,20–28^. Although Zhang and others pointed out in their review article^19^ that this diversity might be attributable more to differences in sampling and analytical methods used than to actual regional differences, we strove to extract meaningful information by comparing our results with those reported from earlier studies.

Detailed estimations of microplastic abundance for Arctic and European snow samples were reported by Bergmann et al*.*^14^ after using a micro-FTIR-imaging with a detection limit of 11 µm. For particles larger than 25 µm, microplastic concentrations were estimated respectively as, on average, 2.7 × 10^2^ particles/L for Arctic snow and 4.9 × 10^3^ particles/L for European snow. These values are of the same order of magnitude as the averaged microplastic abundances observed in our remote snow (6.5 × 10^2^ particles/L) and urban snow (3.2 × 10^3^ particles/L).

The most frequent polymer type we detected was alkyd (Fig. 4a). The presence of alkyd particles was confirmed also in Antarctic snow^27^. Recently, Kameda et al*.*^43^ investigated the abundance, polymer compositions and size distributions of microplastics in the surface waters of the Tsurumi River, Japan with a micro-FTIR-mapping technique providing spatial resolution of 20 µm. They have reported alkyd as a dominant polymer among fine MPs (mostly < 100 µm). Alkyd resins are generally used on a wide and intensive scale in road-marking paints in Japan^43^. Other dominant polymer types examined for this work were EVA, PE, and rubber (Fig. 4a). These compositions were observable in global snow samples^14,19,20,25–27^. Bergmann et al*.*^14^ reported that PA, varnish, rubber, EVA and PE are dominant in European snow. Zhang et al*.*^19^ reviewed that, overall, most plastic polymers detected in snow and ice from cryospheric regions were predominantly polyethylene terephthalate (PET), PA, PE, and rubber. However, PET was not found in this study.

The size distribution of microplastics observed for this study (Fig. 4b) is consistent with general trends reported from earlier works, indicating that MPs in snow (precipitation) are generally smaller than 50 µm. The number of MPs observed generally increases concomitantly with decreasing size^13,14,17^. In terms of morphology, as observed from this work (Fig. 4b), in microplastics from wet and dry deposition, fragments were the dominant shape for fine particles (< 100 µm), whereas fiber shapes were common in larger size calsses^13,44^.

Discussion of this work has been based on a limited number of measurements. Consequently, results might be subject to great chance and uncertainty. Although replicate measurements at the three sites have exhibited that the general trends suggested above are reproducible (Table 1 and Fig. 3), results show some points of variation: several to a few tens of percent difference in microplastic abundance (Table 1) and irregular occurrence of minor components in microplastic composition (Fig. 3). In addition, the sample collection spanned 2 years (Table 1): results might depend also on when the snow samples are collected (difference in precipitation events). For example, using high-resolution spatial and temporal data on plastic deposition and atmospheric back-trajectory analyses, Brahney and others^15^ reported that the deposition rates of MPs under wet conditions significantly correlated to population metrics, as determined by the intersection of the air mass with population centers, although details of temporal dependency of microplastic distributions are still not well known because of the paucity of data. Further studies are necessary to elucidate regional differences in microplastic pollution in snow.

## Supplementary Information

Supplementary Information.

## Data Availability

The datasets used or analyzed for this study are available from the corresponding author on reasonable request.
