# Supplementary material for: Microplastics in snow from protected areas in Hokkaido, the northern island of Japan
Source: Sci Rep. 2023 Jun 19;13:9942. doi: 10.1038/s41598-023-37049-5 (PMC10279708; doi:10.1038/s41598-023-37049-5)
Supplement: Supplementary file 1 — Supplementary Information. [file 41598_2023_37049_MOESM1_ESM.pdf]

Microplastics in snow from protected areas in Hokkaido, the northern island of Japan

Supplementary Information

Hiroshi Ohno[1*], Yoshinori Iizuka[2]

[1]Kitami Institute of Technology, Kitami, Hokkaido, Japan

[2]Institute of Low Temperature Science, Hokkaido University, Sapporo, Hokkaido, Japan

*Corresponding author

E-mail: h_ohno@mail.kitami-it.ac.jp

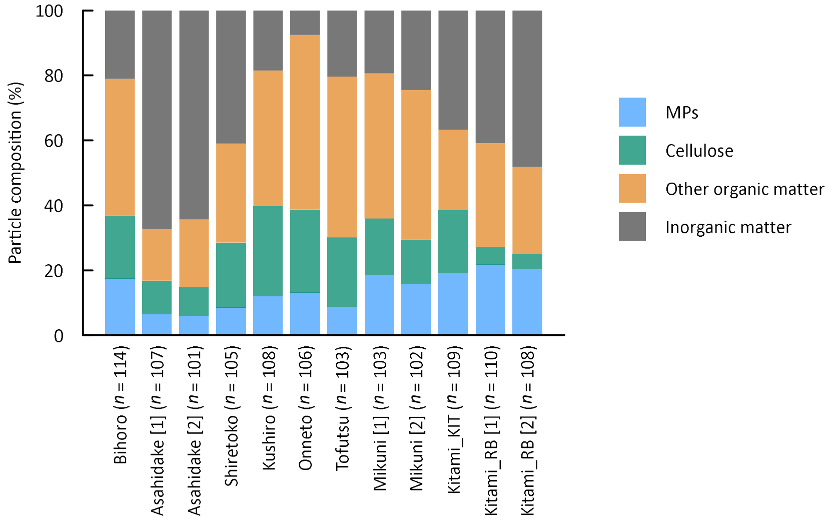

Figure S1. Relative compositions of particles in snow samples identified using micro-FTIR at different locations.

Particle composition at different locations. Compositions of particles from snow samples are shown in Fig. S1. In this figure, particles that exhibit clear IR signals within 2700–3100 cm-1 (C–H stretching modes; e.g. spectra in Fig. 2), but identified as neither MPs nor cellulose are classified into other organic matter, whereas those without C–H stretching bands are categorized as inorganic matter. Microplastics were detected at all locations. The composition ratios of microplastics were approximately 18% at Bihoro Pass, 7% at Asahidake [1], 6% at Asahidake [2], 9% at Shiretoko-Goko Lakes, 12% at Kushiro Shitsugen, 13% at Onneto Lake, 9% at Tofutsu Lake, 18% at Mikuni Pass [1], 16% at Mikuni Pass [2], 19% at Kitami_KIT, 22% at Kitami_RB [1], and 20% at Kitami_RB [2] .
